# The genetics of university success

**DOI:** 10.1038/s41598-018-32621-w

**Published:** 2018-10-18

**Authors:** Emily Smith-Woolley, Ziada Ayorech, Philip S. Dale, Sophie von Stumm, Robert Plomin

**Affiliations:** 1 King’s College London, MRC Social, Genetic and Developmental Psychiatry Centre, Institute of Psychiatry, Psychology & Neuroscience, London, SE5 8AF UK; 2 Department of Speech and Hearing Sciences, University of New Mexico, Albuquerque, 87131 NM USA; 3 Department of Psychological and Behavioural Science, London School of Economics and Political Science, 3rd Floor, Queens House, 55/56 Lincoln’s Inn Fields, London, WC2A 3LJ UK

## Abstract

University success, which includes enrolment in and achievement at university, as well as quality of the university, have all been linked to later earnings, health and wellbeing. However, little is known about the causes and correlates of differences in university-level outcomes. Capitalizing on both quantitative and molecular genetic data, we perform the first genetically sensitive investigation of university success with a UK-representative sample of 3,000 genotyped individuals and 3,000 twin pairs. Twin analyses indicate substantial additive genetic influence on university entrance exam achievement (57%), university enrolment (51%), university quality (57%) and university achievement (46%). We find that environmental effects tend to be non-shared, although the shared environment is substantial for university enrolment. Furthermore, using multivariate twin analysis, we show moderate to high genetic correlations between university success variables (0.27–0.76). Analyses using DNA alone also support genetic influence on university success. Indeed, a genome-wide polygenic score, derived from a 2016 genome-wide association study of years of education, predicts up to 5% of the variance in each university success variable. These findings suggest young adults select and modify their educational experiences in part based on their genetic propensities and highlight the potential for DNA-based predictions of real-world outcomes, which will continue to increase in predictive power.

## Introduction

The difference in earnings between high school and university graduates is estimated at $1 million over the course of the lifetime^1^. However, the difference in earnings varies by the type of university attended^2^, as well as achievement at university^3^. Furthermore, the benefits associated with obtaining a university education extend beyond earnings, to include better health and wellbeing, higher rates of employment and even increased life expectancy^4^. Despite this, little is known about the causes and correlates of differences in university-level outcomes, including entrance into university, achievement at university and the quality of university attended.

Differences in who obtains a university degree and who does not are, at least in part, associated with differences in prior academic achievement. A large literature of quantitative genetic studies shows that achievement in childhood and adolescence are substantially heritable, with 40 to 60% of the individual differences in achievement due to genetic factors^5–8^. However, there are few studies looking at the heritability of academic achievement beyond compulsory education. One twin study, using the same data as in the present study, investigated the heritability of university entrance exams taken at age 18. This study found that the decision to take these exams and students’ average grade were both substantially influenced by genetic factors. Estimates ranged from 50% in the humanities to 60% in science, technology, engineering and mathematics (STEM) subjects^9^. Interestingly, this study found that, although the influence of the shared environment was minimal for average exam grade, it was substantial for the decision to take these exams or not. The shared environment explained almost half of the liability. This suggests that the shared environment, such as family or school, push both members of a twin pair to make the same decisions regarding whether or not to take university entrance exams, but that individual-specific environments influence achievement in university entrance exams. However, the role of shared environmental influence in educational choices at university-level has not yet been studied.

Achievement in high school is highly heritable and stable across development^10^. Indeed, in a longitudinal study using the same sample as in the present study^10^, the heritability of achievement was substantial from age 7 (69%) up to age 16 (61%). In addition, the stability was shown to be driven by genetic effects. For example, 72% of the correlation (*r* = 0.66) between age 7 achievement and achievement at age 16 was shown to be due to additive genetic influences. However, the pattern of stability into university remains unclear. Unlike high school, where there is often a relatively uniform curriculum to follow, university provides students with greater opportunity to carve out their interests and choose environments based on their natural abilities and aptitudes. Because these traits are genetically influenced, the university environments that individuals choose might correlate with their genotype. For example, someone who is naturally talented at math might apply to and attend a university specializing in math, take extra math classes or join a math club. In this way, they have selected environments that correlate with their genetically influenced abilities—a concept known as *gene-environment correlation*^11,12^. Gene-environment correlation has been shown for traits long assumed to be environmental, including life events^13,14^, media use^15^, and occupational status^16^ (for reviews see^17,18^). For this reason, choosing to enroll at university, as well as the quality of the institution, are also likely to show genetic influence.

University quality has been assessed using several different indicators, such as academic reputation, employment prospects, research quality and teaching^19–21^. For example the ‘Complete University Guide’ (https://www.thecompleteuniversityguide.co.uk/league-tables/rankings) takes into account entry standards, student satisfaction, research quality and graduate prospects when ranking UK universities. However, attendance at different quality universities is not random. Because the best quality universities can be highly selective^22^, entrance into university is at least partly dependent on university entrance exam achievement. Therefore, in order to explore the etiology of individual differences in the quality of university students attend it will be important to consider its relationship with prior achievement.

Recent advances in molecular genetics have confirmed a genetic contribution to variance in education-related traits. Genome-wide polygenic scores (GPS), which aggregate the effects of thousands of DNA variants identified through genome-wide association (GWA) studies, can be used to predict educational attainment and achievement. One such GPS which has been found to be predictive of many educationally-relevant traits is a GPS for years of education (*EduYears*^23,24^). The GWA meta-analysis from which this GPS is derived^24^ focused on years of education in a sample of 300,000 individuals. For example, high school completion is counted as 11 years of education, whereas completing a PhD is approximately 20 years. The summary statistics can then be applied to an independent sample to create a GPS. Previously we have shown that *EduYears* GPS explains 2.8% of the variance in academic achievement at age 7, 4.6% at age 12 and 9.1% at age 16 in the current sample^25^. In addition to achievement, *EduYears* GPS has also been used to explore ‘environments’, for example the likelihood of going to university compared to becoming NEET (not in education, employment or training) at age 18^26^, social mobility^27^ and whether students attend selective or non-selective high schools^28^.

In the current study, we use a multi-method approach to investigate the genetics of university success. Capitalizing on both twin and molecular genetic data, we perform the first genetically sensitive study of university success, including: achievement in university entrance exams, enrolment at university, university quality (the ranking of the university in league tables), university quality regressed for prior achievement and achievement at university (final degree grade for the whole sample and separately for STEM and humanities subjects). We also explore the genetic links between these variables using multivariate twin analysis.

## Results

### Phenotypic analyses

Table S1 in the Supplementary Materials lists the sample sizes, means and standard deviations for entrance exam achievement, university quality, university achievement, university enrolment and university quality regressed for university entrance exam achievement separately for males and females and for zygosity groups. Analysis of variance (ANOVA) was performed on each of the continuous university success variables in order to assess the mean effects of sex, zygosity and their interaction. It can be seen from Table S1 that, although there were mean differences between males and females in entrance exam achievement, university quality and university quality regressed for prior achievement, cumulatively they explain less than 1% of the variance. As a result, sex-limitation model fitting was not performed on these variables and for all subsequent analyses, the data were age and sex regressed and van der Waerden transformed^29^. Residuals were retained for twin and genomic comparisons. All twin analyses were conducted on the full sample, combining DZ opposite sex and same sex twin pairs.

#### Sensitivity analyses

Sensitivity analyses comparing entrance exam achievement and university quality between those individuals who did or did not report their final university degree grade were performed. Results indicated that group membership (whether or not a final degree grade was reported) accounted for less than 1% of the variance in entrance exam achievement and university quality suggesting our results were not inflated by missing data (Table S2).

### Intraclass twin correlations

Twin correlations for the university success variables can be found in Table S3 in Supplementary Materials. For all of the measures, MZ twin correlations exceeded those of the DZ twins, suggesting genetic influence. For example the MZ correlations for university entrance exam achievement were approximately 0.70, compared to 0.40 for DZ twins. Rough estimates of additive genetic (A), shared environmental influence (C) and non-shared environmental influence (E) using Falconer’s formula^30^ can be found in Table S3.

### Twin analysis

To investigate the extent to which genetic and environmental factors explained the variance in each of the university success variables, as well as the associations between them, univariate and multivariate genetic analyses were conducted with OpenMx in the R statistical modeling package^31^.

#### Univariate genetic analysis

Univariate twin analyses were performed on the university success variables using structural equation modelling. Here, phenotypic variance in a trait is decomposed into additive genetic (A), shared environmental (C) and non-shared environmental (E) influences. For further information on the univariate twin analysis, see the Method section. We tested full ACE models, as well as nested models (AE and CE) and found that, for university entrance exams and university quality, ACE models fit the data best (Tables S4 and S5). For university achievement and university quality regressed for university entrance exams, AE models’ fit the data best (Tables S6 and S7). For university enrolment, we fit a liability threshold model (Table S8), which decomposes the liability of going to university or not into A, C and E.

**Figure 1:**
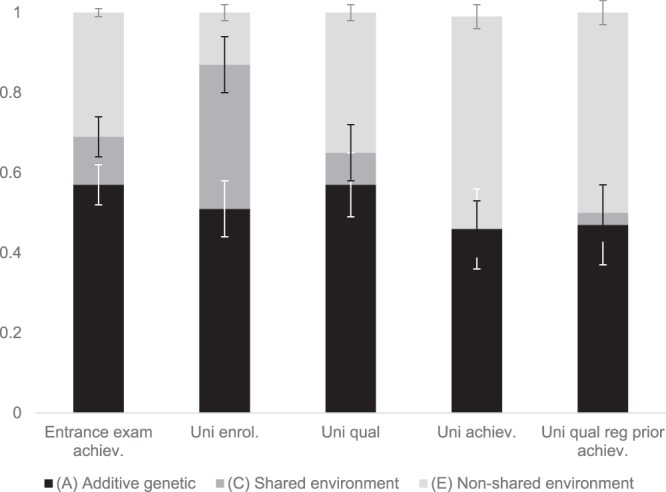
Model-fitting results and 95% confidence intervals for additive genetic (A), shared environment (C), and non-shared environment (E) components of variance for entrance exam achievement, university enrolment, university quality, university achievement and university quality regressed for entrance exam achievement.

Figure 1 shows the variance in each of the university success markers that can be attributed to A, C and E. Table S9 gives these estimates along with their 95% confidence intervals. All of the measures were substantially genetically influenced, with additive genetics accounting for between 46% and 57% of the variance. These estimates were in line with the intraclass twin correlations (Table S3). The heritability of achievement decreased from entrance exam achievement (57%) to university achievement (46%), as did the influence of the shared environment, which was no longer significant for university achievement. University enrolment showed the most shared environmental influence (36%). University quality was highly heritable (57%), with most of the remaining variance explained by the non-shared environment.

Interestingly, the measure of university quality continued to be substantially heritable (47%) even after we accounted for entrance exam achievement. This suggests that the high heritability of university quality reflects more than just prior achievement.

#### Multivariate genetic analysis

To test the genetic and environmental links between the variables, multivariate genetic analyses were also conducted. To find the best-fitting model, we tested three multivariate designs: correlated factors, common pathway and independent pathway models, and compared their fit statistics (Table S10). Correlated factors was the best-fitting model and the results are presented in this form.

Genetic, shared environmental and non-shared environmental correlations between university success variables can be found in Table S11. As indicated by the genetic correlations (Table S11, part a), there was a high degree of genetic correlation between entrance exam achievement and university quality (r_g_ = 0.76). Furthermore, there was also a moderate degree of genetic correlation between entrance exam achievement and university achievement (r_g_ = 0.49). Genetic correlations were weaker between entrance exam achievement and university achievement (r_g_ = 0.27). Turning to shared environmental correlations (Table S11, part b), there was a high shared environmental correlation between entrance exam achievement and university quality (r_c_ = 0.81). Shared environmental correlations between entrance exam achievement and university achievement and between university quality and university achievement were weaker (r_c_ = 0.35 and 0.27 respectively). Non-shared environmental correlations (Table S11, part c) were moderate between entrance exam achievement and university quality (r_e_ = 0.35), however they were mainly non-overlapping for entrance exam achievement and university achievement (r_e_ = 0.03) and between university quality and university achievement (r_e_ = 0.09).

### Polygenic score analysis

**Figure 2:**
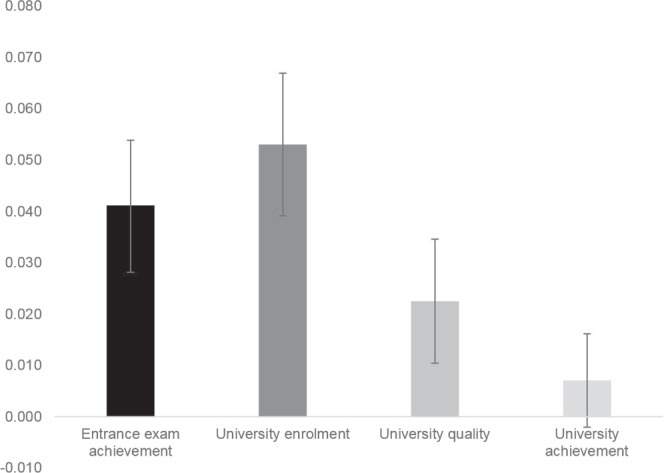
Variance explained (R^2^) and 95% confidence intervals by *EduYears* genome-wide polygenic score for each of the university success variables.

To investigate the extent to which SNPs associated with years of education (*EduYears*) predicted the university success variables, we created a genome-wide polygenic score (GPS) and correlated it with our variables (for more information on how this GPS was created, see the Method section). The *EduYears* GPS significantly predicted university success variables, explaining 4% of the variance in entrance exam achievement, 5% in university enrolment, 2% in university quality and 0.7% in university achievement (Fig. 2, for values, see Table S12). Furthermore, there was no difference in prediction of *EduYears* GPS between achievement in humanities subjects compared to achievement in STEM subjects (*z* = 1.08, *p* = 0.28; see Table S13).

## Discussion

Our results represent the first genetically sensitive exploration of success at university using twin and genomic data. Twin analysis revealed substantial heritability for all university success measures, including university entrance exam achievement (57%), the choice to study at university (51%), the quality of university attended (57%) and achievement at university (46%). In addition to twin analysis, we also found evidence for genetic influence using DNA alone. Indeed, a genome-wide polygenic score (GPS) for adult educational attainment^24^ explained up to 5% of variance in the university success variables. Taken together, these results highlight that the appetite and aptitude young adults have for higher education is, in part, genetically influenced.

Finding genetic influence on success in university extends a vast literature on education and genetics^5,32^. The present results show for the first time that genetic influence on educational achievement continues to university. This is in line with twin estimates in earlier school years. For example, one study^9^ using the same sample found that at age 18, the heritability of achievement in different subjects ranged from 23–82%. Interestingly, the substantial influence of the shared environment on educational achievement during the early school years tapers off at university. Indeed, shared environmental influences account for up to 20% of the variance in the compulsory school years^33^, but make a non-significant contribution to variance in achievement at university. One explanation for this pattern of results is that in the early school years children’s environments are largely the same across multiple life domains, for example siblings go to the same school, have many of the same friends, and spend much of their time at home under substantial parental influence. By contrast, young adults have more freedom of choice in their education, in terms of the subjects they take, the extracurricular activities they engage in and how they spend their time. This increase in choice leads to greater genetic influence and decreased shared environmental influence across development. We see this same developmental decrease in the influence of the shared environment for other educationally-relevant traits such as intelligence^34^. This suggests that as children gain more freedom to choose their environments, they increasingly select environments that correlate with their genotype.

An exception to this developmental decrease in shared environmental influence pertains to decisions about whether to continue in education. For example, shared environmental influences account for nearly 40% of the variance in the choice of whether or not to take university entrance exams^9^ and the choice of whether or not to pursue a university degree, yet shared environmental influence is not evident for university achievement. It is possible that families and schools influence educational choices to a greater extent than educational achievement.

Multivariate genetic analyses indicated a substantial genetic correlation between university entrance exam achievement and university quality (76%). These results support the generalist genes hypothesis of cognitive traits^35^, which suggests that most genetic influences are shared across learning abilities and therefore educationally relevant genes will influence a range of associated traits, for example intelligence^25^, SES^25^ and now, university success. Along with genetic correlations, we also found moderate shared environmental correlations between university success variables (r_c_ = 27–81%). Non-shared environmental influences were mainly uncorrelated (r_e_ = 3–35%). This suggests that unique environmental factors that contribute to variance in university success are idiosyncratic and time specific and do not contribute to effects across compulsory and higher education.

Although we found moderate twin heritability estimates for university achievement, the polygenic score prediction of this trait was small in magnitude, only predicting 0.7% of the variance. Furthermore, even when we split university achievement into two groups: STEM-related subjects and humanities subjects, we did not find any differences in *EduYears* GPS prediction between subjects. This suggests that even within subject field, the GPS is not discriminative of achievement. In contrast, *EduYears* GPS explains 9% of the variance in achievement at age 16^25^. There are several possible reasons for this paradox. First, a polygenic score based on years of education might be less discriminative for individuals who have all obtained a university degree. Second, examinations at university level are not standardized, which means that results may be less comparable between universities; a first-class degree at an elite university will be weighted the same as one from a lower-level university. This interpretation is supported by the low MZ correlations for university achievement (0.30), compared to the MZ correlations for the other university success measures (0.50–0.69). Such coarseness in measurement may render the *EduYears* polygenic score less capable of predicting individual differences. Finally, it is possible that getting into university and achievement at university are predicted by different heritable traits. Indeed even standardized tests such as the Scholastic Aptitude Tests (SATs) that are widely used for college admittance in the United States are poor predictors of both four and six-year university graduation rates after admittance^36^. Future studies using multivariate genetic modeling can test this differential heritability hypothesis.

In contrast to the results for university achievement, *EduYears* polygenic score predicted variance in both the decision to attend university, as well as the choice of which university to attend. These results are in line with our twin analysis demonstrating substantial genetic influence on educational choices. Both the decision of whether or not to go to university, and which university to attend, are influenced by an individual’s educational qualifications, which we know are substantially heritable^37^. However, even once we controlled for prior academic achievement, the quality of university attended was still considerably heritable (47%). This is likely because, in addition to getting the right grades, there are other heritable factors which influence both the decision to go to university, as well as the decision to go to one university over another, for example socio-economic status, friendships, secondary school quality and parental involvement with students’ learning^38^.

The present study benefited from a large sample size of over 3,000 twin pairs and over 3,000 genotyped individuals, as well as a multi-method approach. However, our results must be considered in light of limitations of current DNA methods, in addition to the general limitations of the twin method (Knopik *et al*., 2017).

The *EduYears* GPS explains only a fraction of the known high heritability of educationally relevant traits as estimated from twin studies. This is because GPS are derived from GWA studies that are limited to estimating additive genetic effects from common SNPs present on DNA arrays or variants in linkage disequilibrium. For this reason, GPS will underestimate genetic influence to the extent that non-additive effects or rare variants contribute to its heritability. However, there has been limited success for detecting non-additive variation in GWA studies. Potential reasons for this are 1) non-additive effects do not appear to make up a large fraction of the total genetic variation, as identified by twin studies and 2) because effects are likely very small, large sample sizes would be needed^39^. SNP-based estimates of heritability, which have these same limitations, represent the current upper limit for GPS prediction. Although we were underpowered to calculate SNP-based heritability estimates in the present study, our data collection is ongoing, and we plan to explore SNP-based estimates for university success in the future. As the so-called missing-heritability gap closes, GPS predictions will improve and will increasingly be used as an index of genetic influence on complex human behavior^40^.

Despite this limitation of our molecular genetic analysis, this study represents the first genetically informative study of university success. We show that genetic influences on education trajectories are pervasive and cumulative into young adulthood and affect both appetite for education and aptitude for learning.

## Method

### Participants

Participants were drawn from the UK-representative Twins Early Development Study (TEDS). TEDS is a multivariate and longitudinal birth cohort study that recruited over 15,000 twin pairs born in England and Wales between January 1994 and December 1996. The representativeness of the TEDS sample has been assessed longitudinally and is described in further detail elsewhere^41^. The representativeness of the TEDS twins to the UK population has been demonstrated in infancy, early childhood, middle childhood, adolescence^5,41^ and in early adulthood for each of our university success variables^42,43^ (Department for Education, 2016). For example, percentages of TEDS participants were similar to UK national averages for enrolling in university (56% vs 49%) and obtaining a first class degree (33% vs 26%). In addition, the genotyped sub-sample is representative of the UK for gender, parental education and rates of employment for both mothers and fathers^25^.

All analyses were conducted on participants without severe neonatal problems. Ethical approval for this study was received from King’s College London Ethics Committee and all methods were carried out in accordance with the relevant guidelines and regulations. All participants provided written informed consent.

### Twin sample

Zygosity was based on parent reports of twin differences during childhood, which is over 95% accurate when compared to DNA testing^44^. For cases where zygosity was unclear, DNA testing was conducted. After exclusions, data on entrance exam achievement were available for 4,698 twin pairs (9,407 individuals), of which 1,671 were monozygotic (MZ) twin pairs, 1,519 were dizygotic (DZ) same-sex twin pairs and 1,508 were DZ opposite-sex twin pairs. Data on university enrolment were available for 5,143 twin pairs (10,288 individuals), of which 1,812 were MZ twin pairs, 1,678 were DZ same-sex twin pairs and 1,653 were DZ opposite-sex twin pairs. Data on university quality were available for 3,034 twin pairs (6,091 individuals), of which 1,057 were MZ twin pairs, 963 were DZ same-sex twin pairs and 1,014 were DZ opposite-sex twin pairs. Finally, data on university achievement were available for 1,590 twin pairs (3,219 individuals), of which 608 were MZ twin pairs, 481 were DZ same-sex twin pairs and 501 were DZ opposite-sex twin pairs

### Genomic sample

The TEDS sample includes a genotyped subsample of unrelated individuals (i.e., one member of a twin pair). Genotypic analyses were restricted to participants of European decent, as ascertained by TEDS questionnaire data at first contact when the twins were aged 2. Standard principal component analyses were used to confirm the European ancestry of the sample. Here we regressed the GPS on the first 10 principal components and used the residuals in all subsequent analyses. This procedure controls for population stratification, which is the systematic difference in allele frequencies observed in subpopulations of individuals of different ancestry. Genomic data for creating genome-wide polygenic scores (GPS) were available for 3,501 individuals with data on entrance exam achievement, 3,774 individuals with data on university enrolment, 2,251 individuals with data on university quality and 1,291 individuals with data on university achievement. This genotyped sample is representative of UK census data on education and socioeconomic related phenotypes for families with children, for example the percentage whose parents went on to further education and parental employment^25^.

DNA was genotyped using Illumina HumanOmni ExpressExome-8v1.1 arrays (Institute of Psychiatry, Psychology and Neuroscience Genomics & Biomarker Core Facility, London, United Kingdom) or Affymetrix GeneChip 6.0 DNA arrays (Affymetrix, Santa Clara, CA). The sample with genotype data consisted of 5,825 individuals (2,698 genotyped with Illumina and 3,127 genotyped with Affymetrix arrays). Genome wide genotypes from the two arrays were separately imputed using the Haplotype Reference Consortium^45^ and the imputation software Minimac3 1.0.13^46^, which are available from the Michigan Imputation Server (https://imputationserver.sph.umich.edu). A series of quality checks were performed before merging data from the two arrays imputation (e.g. array effects, allele frequencies by imputation quality). For the present analyses, we limited our analyses to variants genotyped or imputed at info >0.95 on both arrays, and with Hardy Weinberg Equilibrium test *p*-value > 10^−5^.

Linkage disequilibrium (LD) refers to the non-random association of alleles at different loci. When calculating polygenic scores using PRSice those markers in high LD are removed or ‘pruned’ as correlated variants can represent non-independent association signals that if ignored can overweight GPS in favour of loci in high LD. Stringent pruning resulted in the exclusion of eight genomic regions in high linkage disequilibrium (R^2^ > 0.1 cutoff within a 250-kb window). To ensure that only genome wide effects were detected, we performed a principal components analysis to correct for possible stratification using a subset of 40,745 autosomal single-nucleotide polymorphisms (SNPs) that remained after we applied our quality-control criteria and that overlapped between the two genotyping arrays.

## Measures

The present study included individuals with data available on measures of ‘university success’, as described below. These measures were obtained from the twins at ages 18 and 22 using paper and online questionnaires, as well as a mobile phone application. For sample sizes across each of the measures, please see Table S1.

### University entrance exams variables

In the UK, getting into university depends on achievement on the General Certificate of Education Advanced Level, or ‘A-levels’ (https://www.ucas.com/ucas/undergraduate/getting-started/ucas-undergraduate-entry-requirements). A-levels are a two-year school leaving qualification offered at the end of compulsory education at age 16, when students are allowed to decide, for the first time, whether they want to continue formal education. In addition to choosing whether they wish to continue education, students are also able to pick what they want to study, with students typically completing three A-levels in a variety of subjects. If students only complete one out of the two A-level years they are awarded an AS-level qualification.

#### Entrance exam achievement

A-levels are graded from E, the minimum pass grade to A*, the best possible grade. For university entry, these grades are converted into Universities and Colleges Admissions Service (UCAS) points. Following this point system, achieving an A* at A-level is awarded 56 points, whereas an E is awarded 16 points. If students only complete an AS-level qualification, UCAS points are adjusted accordingly, with an A* grade at AS-level awarded 20 points and an E grade at AS-level awarded 6 points (for further information on UCAS points, see: https://www.ucas.com/ucas/tariff-calculator). Data on UCAS point scores were available for our sample through the National Pupil Database: https://www.gov.uk/government/collections/national-pupil-database.

### University variables

#### University enrolment

Data on whether or not twins chose to attend university was collected via questionnaire at age 18. This questionnaire was designed to assess post-18 destinations. Choosing to attend university was treated as a dichotomous variable, where 1 indicated the choice to pursue university and 0 indicated any other post compulsory education destination, such as going into employment, training or unemployment. Approximately 57% of our sample reported accepting a place at university, which is similar to the UK average^42^.

#### University quality

For those who indicated that they were attending university, we also asked for the name of the university they attended. We used this information to create a university quality measure by ranking the universities in order based on the UK university league tables in 2014 (the year that the majority of the sample applied to university)^47^. This ranking system takes into account the entry standards of the university, the average UCAS points of students at the university, research output, and graduate prospects. According to this ranking system the University of Cambridge was at the top, and East London University was at the bottom, with 124 universities in total.

To explore the unique contribution of our university quality variable beyond the effect of previous educational achievement, we regressed university quality on entrance exam achievement.

#### University achievement

At age 22 (M = 22, SD = 0.85), we contacted twins about their higher education choices, including whether or not they had completed an undergraduate degree and what grade they obtained. Undergraduate university degrees were graded from 1 (the lowest possible pass) to 5 (a first-class degree, the highest possible pass). Of those individuals who indicated that they were attending university at age 18 (N = 5,833), over half provided their final university grade at age 22 (N = 3,219). To check whether there were any achievement or university quality differences between those who reported their final degree grade and those who did not, we performed sensitivity analysis. Here, a t-test compared entrance exam achievement and university quality between those individuals who stated they were attending university but did or did not report their final university degree grade (see Supplementary Table S2).

#### Degree subject

At age 22, we also asked twins to choose a category that best described their degree subject (see Table S13 for a list of categories and sample sizes). We classed those people who selected ‘natural sciences’, ‘mathematics/statistics’, ‘medicine/veterinary’, ‘engineering’, ‘technology/design’ or ‘computing/IT’ as STEM and those who took ‘social sciences’, ‘arts’, ‘humanities’, ‘languages’ or ‘law’ as Humanities. Descriptive statistics for STEM vs Humanities degree subjects can be found in Table S13.

A correlation matrix of all of the variables included in the current study can be found in Supplementary Figure S1.

## Statistical Analyses

### Twin analyses

Univariate twin analyses were used to compare twin similarity on university entrance examinations, university quality and university achievement between MZ twin pairs who share 100% of their genetic material and DZ twin pairs who share on average 50% of the genetic material that can differ between individuals (segregating alleles). Twin analyses were used to estimate the proportion of variance in university entrance examinations, university quality and university achievement that can be attributed to genetic and environmental factors^33^. The genetic contribution to phenotypic variance is referred to as heritability (A) and is narrowly defined as the proportion of individual differences in a population that can be attributed to additive effects of inherited DNA differences between individuals. We can roughly estimate ‘A’ by doubling the difference between MZ and DZ intraclass correlations on a trait. The environmental contribution to phenotypic variance includes those non-inherited influences that are shared (C) and unique (E) to twins growing up in the same home. The C component refers to those environmental factors that contribute to twin similarity and can be calculated by subtracting ‘A’ from the MZ twin correlations. The E component captures environmental experiences unique to the individual as well as measurement error and is measured by deducting the A and C components from unity.

The ACE estimates for twin analyses can be calculated more precisely using structural equation modelling (SEM) with the OpenMX software package^31^, which also provides confidence intervals around the estimates.

Statistical approaches for analyzing twin data are described elsewhere^48,49^. Briefly, SEM leverages the different sources of sibling similarity and differences to make inferences on the etiology of observable traits. SEM tests hypotheses about relations among observed phenotypic correlations and latent genetic and environmental factors by modeling the observed covariance between MZ and DZ twin pairs on the phenotype. Here, model parameters are estimated by minimising a goodness-of-fit statistic that seeks to obtain the smallest possible discrepancy between the model and the observed data. A likelihood ratio chi-square statistic (χ^2^) is then used to measure the goodness of fit of the tested model relative to a perfectly fitting (saturated) model. A significant (*p* < 0.05) χ^2^ when comparing the tested model to the saturated model means that the model provides a poor fit to the data and can be rejected, while a non-significant χ^2^ means that the model is consistent with the data. In the present analyses, we tested a series of nested models to determine whether the components, A, C and E, are significantly greater than zero. With each test we assessed whether the fit of the simpler, nested model was significantly worse than that of the full model, with preference for a simpler more parsimonious explanation of the observed data. Details of each model tested are presented in the Supplementary Material.

Finally, a liability threshold model (LTM) was used to compute ACE estimates for university enrolment. The LTM is an extension of the classic univariate twin analyses, used for dichotomous variables, for example, in case-control studies comparing individuals with diagnoses to those without. Here, binary variables are assumed to represent an unobserved normal distribution^31^ and twin tetrachoric (rather than intraclass) correlations are compared to index relative genetic and environmental contribution to the liability. Similar to the univariate model, greater MZ compared to DZ correlations can be used to estimate the ACE components to the liability variance. Sub-model comparisons for the LTM compared a fully saturated model with a constrained model (Sub 1) where thresholds were constrained across twin 1 and twin 2 within zygosity groups and a second model (Sub 2) where thresholds were equated across twin pairs and zygosity. Similar to assessment of univariate and multivariate SEM described above, model fit statistics were used to isolate the most parsimonious fit to the data.

Multivariate model fitting is an extension of univariate twin analyses that relies on cross-twin cross-trait correlations to decompose phenotypic covariance between multiple traits into genetic and environmental components of covariance. The correlated factor model, which was found to be the best fit to the data, was used to estimate A, C, and E correlations between our continuous university success variables. This model assumes each variable is influenced by a set of genetic, shared and non-shared environmental factors that are allowed to correlate with each other through r_a_, r_c_ and r_e_. Model fit statistics are provided in the Supplementary Material (Table S10).

#### Genomic analyses

A genome-wide polygenic score (GPS) was derived from summary statistics from a published genome-wide association (GWA) study of years of education^24^. The GPS serves as an individual-specific genetic prediction derived directly from DNA and is calculated by summing genotypic values for each trait-associated single nucleotide polymorphism (SNP) weighted by its association in the GWA study sample. A GPS was calculated for each of the unrelated, genotyped individuals in the TEDS sample using PRSice^50^. Here, PRSice performs a regression analysis to test for association between GPS and each of our university success outcomes. We used the high-resolution scoring option in PRSice which calculates GPS at a large number of *p*-value thresholds, ranging from 0.001 to 1 (increments of 0.001) in the GWA study results. For all university success variables, the most predictive threshold was 0.05 (i.e., including all GWA study identified SNPs with *p-*value up to 0.05), which included 19,415 SNPs. The results for the 0.05 threshold are reported in the manuscript while results for each of the other tested thresholds is provided in Figure S2.

The difference between genetic estimates from twin and polygenic score analysis should be noted. Additive genetic influences (A) in twin models include additive genetic effects of any DNA sequence differences. In contrast, the polygenic score prediction only includes the additive effects of common SNPs in DNA arrays that have been linked to the target trait of a GWA study. Therefore, it is to be expected that GPS will account for a small amount of variance compared to the sum total of all genetic effects (heritability) estimated in twin studies.

Regression models were then used to estimate the proportion of variance in the continuous (R^2^) or dichotomous (Nagelkerke R^2^) university success variables that can be explained by variance in individuals’ GPS. Furthermore, to test for potential correlation differences between *EduYears* GPS predictions of university degree grades for STEM versus humanities subjects, we performed Fisher’s *r*-to-*z* transformations.

## Electronic supplementary material

Supplementary Material

## Data Availability

For information on data availability, please see the Twins Early Development Study data access policy. This can be found at: http://www.teds.ac.uk/research/collaborators-and-data/teds-data-access-policy.
