# Supplementary material for: The genetics of university success
Source: Sci Rep. 2018 Oct 18;8:14579. doi: 10.1038/s41598-018-32621-w (PMC6194053; doi:10.1038/s41598-018-32621-w)
Supplement: Supplementary file 1 — Supplementary Material [file 41598_2018_32621_MOESM1_ESM.docx]

**Supplementary Online Material**

**The genetics of university success**

Emily Smith-Woolley, Ziada Ayorech, Sophie von Stumm, Philip S. Dale, Robert Plomin

| **Supplementary Tables** | **Page** |
| --- | --- |
| **Table S1**. Mean (standard deviation) of each of the university success variables for the total sample and across gender and zygosity. | 2 |
| **Table S2.** Sensitivity analyses comparing entrance exam achievement and university quality between those individuals who did or did not report their final university degree grade | 3 |
| **Table S3**. Twin correlations (sample size) for university success variables across gender and zygosity. | 4 |
| **Table S4.** Univariate model fitting results for entrance exam achievement | 5 |
| **Table S5.** Univariate model fitting results for university quality | 6 |
| **Table S6.** Univariate model fitting results for university achievement | 7 |
| **Table S7.** Univariate model fitting results for university quality independent of entrance exam achievement | 8 |
| **Table S8.** Liability threshold model fitting results for university enrolment | 9 |
| **Table S9**. Twin *ACE* estimates indicating the proportion of variance in university success variables attributable to genetic and environmental factors. | 10 |
| **Table S10.** Multivariate model fitting results for university entrance exam achievement, university quality and university achievement. Comparing correlated factors, independent pathway and common pathway models. | 11 |
| **Table S11.** Genetic, shared environmental and non-shared environmental correlations between university success measures | 12 |
| **Table S12.** Polygenic score prediction (R²) of university success variables with 95% confidence intervals | 13 |
| **Table S13.** Sample sizes and EduYears GPS prediction (R²) for STEM and humanities subjects | 14 |
|  |  |
| **Supplementary Figures** | **Page** |
| **Figure S1.** Correlations between each of the university success variables. | 15 |
| **Figure S2.** Polygenic score prediction for each of the university success variables at each of *pvalue* thresholds tested using PRSice. | 16 |

|  | |  | | | **Means** | | | | | | | | | | **ANOVA** | | | | | |
| --- | --- | --- | --- | --- | --- | --- | --- | --- | --- | --- | --- | --- | --- | --- | --- | --- | --- | --- | --- | --- |
| **Uni success** | ***N*  whole** | | ***N*  *GPS*** | **Whole  sample** | | **Male** | **Female** | **MZm** | **DZm** | **MZf** | **DZf** | **Dzos** | **DZss** | **Sex** | | **Zyg** | **Sex x Zyg** | **R^2^** | |  |
| Entrance exam achiev. | 9407 | | 3501 | 322.73  (129.91) | | 310.96 (136.86) | 332.1 (123.32) | 315.04 (137.92) | 308.05 (138.86) | 329.15 (125.33) | 334.28 (125.71) | 322.41 (126.41) | 322.4 (132.46) | 61.89** | | 0.18 | 10.13** | <0.01 | |  |
| University quality | 6091 | | 2251 | 45.97 (32.81) | | 44.00  (32.11) | 47.44 (33.25) | 44.12 (32.03) | 43.69 (31.98) | 47.81 (33.25) | 46.43 (32.91) | 46.27 (33.07) | 45.25 (32.53) | 16.43** | | 0.44 | 2.79** | <0.01 | |  |
| University achiev. | 3219 | | 1291 | 4.21 (0.66) | | 4.22 (0.67) | 4.21 (0.65) | 4.27 (0.67) | 4.27 (0.46) | 4.23 (0.64) | 4.19 (0.68) | 4.21  (0.65) | 4.21 (0.68) | 0.06 | | 0.14 | 1.05 | <0.01 | |  |
| University enrolment | 10288 | | 3774 | Y= 57% | | Y=52% | Y=61% | Y=51% | Y=50% | Y=61% | Y=60% | Y=58% | Y=55% |  | |  |  |  |  |  |
| Reg Uni qual | 5918 | | 2179 | 0.00  (1.00) | | -0.09  (0.95) | 0.07  (1.03) | -0.06  (0.91) | -0.10  (0.96) | 0.10  (1.04) | 0.04  (1.03) | -0.02  (1.01) | -0.02  (1.00) | 9.53* | | 0.95 | 0.20 | <.001 |  |  |

**Table S1.** Mean (standard deviation) of each of the university success variables for the total sample and across gender and zygosity.

***Note:*** *N whole* = total sample size (both twins in a pair) after exclusions; *N GPS* = total sample size for the genotyped sample; *MZ*= monozygotic; *DZ*= dizygotic; *m*= male; *f*= female; *os*= opposite sex; Entrance exam achiev = university entrance exam grades calculated by converting achievement scores on the General Certificate of Education Advanced Level into Universities and Colleges Admissions Service (UCAS) points; University Quality= quality of university attended based on the UK university league tables in 2014; University Achiev= grade achieved at university, graded from 1 (a pass, the lowest possible pass) to 5 (a first-class degree, the highest possible pass); University enrolment = proportion of sample that either did or did not go on to university Y/N (no further analysis was done with this variable as it is dichotomous); Y*=* total number of individuals who went on to university in per cent; Reg uni qual. = saved standardised residuals following regression of university entrance exam achievement on university quality. Analyses of variance (ANOVA) performed on raw data from one randomly selected twin per pair to test the effect of sex, zygosity and their interaction. Results = *F* statistic; * = p<.05; ** = p<.01; R^2^= proportion of variance explained by sex, zygosity and their interaction.

**Table S2.** Sensitivity analyses comparing entrance exam achievement and university quality between those individuals who did or did not report their final university degree grade

|  | **Data not present**  **Mean (*SD*)** | **Data present**  **Mean (*SD*)** | ***t*** | ***df*** | **Mean difference** | ***R²*** |
| --- | --- | --- | --- | --- | --- | --- |
| Entrance exam achievement | 0.26 (0.86) | 0.46 (0.99) | -8.87** | 5550 | -0.21 | 0.01 |
| University quality | 0.03 (0.87) | -0.07 (1.02) | 3.56** | 5445 | 0.10 | <0.01 |

***Note:*** t test comparing achievement and university quality differences between individuals who did or did not report their final university degree grade but previously stated that they were attending university. We found significant mean differences due to large sample sizes, however only 1% of variance was explained by group membership; ** p <.001.

**Table S3**. Twin correlations (sample size) for university success variables across gender and zygosity.

|  | **Twin correlations** | | | | | | | |  | **Falconer’s formula** | | |
| --- | --- | --- | --- | --- | --- | --- | --- | --- | --- | --- | --- | --- |
|  | **MZ full** | **DZ full** | **MZm** | **DZm** | **MZf** | **DZf** | **Dzos** | **DZss** |  | **A** | **C** | **E** |
| Entrance exam achiev. | 0.69 (N=3,339) | 0.38 (N=6,068) | 0.72  (N= 1,355) | 0.40  (N=1,377) | 0.67  (N=1,984) | 0.42  (N=1,663) | 0.35  (N=3,028) | 0.41  (N=3,040) |  | 0.62 | 0.07 | 0.31 |
| Uni. enrolment* | 0.66  (N=3,591) | 0.42  (N=6,697) | 0.70  (N= 1,576) | 0.43  (N=1,579) | 0.62  (N=2,015) | 0.44  (N=1,785) | 0.39  (N=3,333) | 0.44  (N=3,364) |  | 0.48 | 0.18 | 0.34 |
| Uni. quality | 0.65  (N=2,130) | 0.34  (N=3,961) | 0.69  (N= 842) | 0.41  (N=835) | 0.61  (N=1,288) | 0.42  (N=1,103) | 0.28  (N=2,023) | 0.42  (N=1,938) |  | 0.62 | 0.03 | 0.35 |
| Uni. achievement | 0.30  (N=1,222) | 0.10  (N=1,997) | 0.29  (N= 370) | 0.04  (N=310) | 0.33  (N=852) | 0.09  (N=675) | 0.12  (N=1,012) | 0.08  (N=985) |  | 0.30 | 0.00 | 0.70 |
| Uni. quality regressed for prior achievement | 0.50  (N=1,636) | 0.26  (N=2,550) | 0.51  (N=648) | 0.34  (N=528) | 0.48  (N=988) | 0.31  (N=740) | 0.15  (N=1282) | 0.37  (N=1268) |  | 0.48 | 0.02 | 0.50 |

***Note:*** MZ = monozygotic; DZ = dizygotic; m= male; f= female; os= opposite sex; ss= same sex; *N*=sample size (individuals) after exclusions; * = university enrolment was a dichotomous variable (university yes or no), therefore we calculated tetrachoric correlations. It is possible to calculate rough estimates of additive genetic influence (A), shared environmental influence (C) and non-shared environmental influence (E) by using Falconer’s formula. A = 2 * (rMZ - rDZ); C = rMZ – A; E = 1 – rMZ.

**Table S4.** Univariate model fitting results for entrance exam achievement

| **Model** | **ep** | **-2LL** | **df** | **AIC** | **∆ LL** | **∆ df** | ***p*** |
| --- | --- | --- | --- | --- | --- | --- | --- |
| 1. Full saturated | 10 | 52571.27 | 19506 | 13559.27 | - | - | - |
| 2. Constrained means across twin order | 8 | 52571.27 | 19508 | 13555.27 | 4.79e-09 | 2 | 1.000 |
| 3. Constrained means and variances across twin order | 6 | 52571.27 | 19510 | 13551.27 | 8.73e-11 | 4 | 1.000 |
| 4. Constrained means and variances across twin order and zygosity | 4 | 52574.85 | 19512 | 13550.85 | 3.75 | 6 | 0.730 |
| ACE model | 4 | 52574.85 | 19512 | 13550.85 | 3.57 | 6 | 0.73 |
| AE model | 3 | 52598.27 | 19513 | 13572.27 | 23.42 | 1 | 1.30-06 |
| CE model | 3 | 52976.81 | 19513 | 13950.81 | 401.96 | 1 | 2.06e-89 |

***Note:*** ep= estimated parameters; -2LL= -2 log-likelihood; df= degrees of freedom; AIC= Akaike’s information criteria; **∆**LL= change in log-likelihood; **∆**df= change in degrees of freedom. The ACE model fit the data best. Estimated parameters in the full model: 4 means (MZ twin 1 mean, MZ twin 2 mean, DZ twin 1 mean, DZ twin 2 mean), 4 standard deviations ‘SD’ (MZ twin 1 SD, MZ twin 2 SD, DZ twin 1SD, DZ twin 2 SD), 2 within twin correlations (MZ and DZ). This Saturated model is then constrained across twin means, variances, twin order and zygosity (4 estimated parameters) to test whether twin modelling assumptions have been met.

**Table S5.** Univariate model fitting results for university quality

| **Model** | **ep** | **-2LL** | **df** | **AIC** | **∆ LL** | **∆ df** | ***p*** |
| --- | --- | --- | --- | --- | --- | --- | --- |
| 1. Full saturated | 10 | 33463.26 | 12248 | 8967.26 | - | - | - |
| 2. Constrained means across twin order | 8 | 33463.26 | 12250 | 8963.26 | -1.84e-08 | 2 | 1.000 |
| 3. Constrained means and variances across twin order | 6 | 33463.26 | 12252 | 8955.44 | -2.01e-08 | 4 | 1.000 |
| 4. Constrained means and variances across twin order and zygosity | 4 | 33463.26 | 12254 | 8955.44 | 0.18 | 6 | 1.000 |
| ACE model | 4 | 33463.44 | 12254 | 8955.44 | 0.18 | 6 | 1.000 |
| AE model | 3 | 33468.11 | 12255 | 8958.11 | 4.67 | 1 | 0.031 |
| CE model | 3 | 33651.62 | 12255 | 9141.62 | 188.18 | 1 | 7.94e-43 |

***Note:*** ep= estimated parameters; -2LL= -2 log-likelihood; df= degrees of freedom; AIC= Akaike’s information criteria; **∆**LL= change in log-likelihood; **∆**df= change in degrees of freedom. The ACE model fit the data best. Estimated parameters in the full model: 4 means (MZ twin 1 mean, MZ twin 2 mean, DZ twin 1 mean, DZ twin 2 mean), 4 standard deviations ‘SD’ (MZ twin 1 SD, MZ twin 2 SD, DZ twin 1SD, DZ twin 2 SD), 2 within twin correlations (MZ and DZ). This Saturated model is then constrained across twin means, variances, twin order and zygosity (4 estimated parameters) to test whether twin modelling assumptions have been met.

**Table S6.** Univariate model fitting results for university achievement

| **Model** | **ep** | **-2LL** | **df** | **AIC** | **∆ LL** | **∆ df** | ***p*** |
| --- | --- | --- | --- | --- | --- | --- | --- |
| 1. Full saturated | 10 | 18610.94 | 6652 | 5306.94 | - | - | - |
| 2. Constrained means across twin order | 8 | 18610.94 | 6654 | 5302.94 | 2.31e-08 | 2 | 1.000 |
| 3. Constrained means and variances across twin order | 6 | 18610.94 | 6656 | 5298.94 | 1.13e-10 | 4 | 1.000 |
| 4. Constrained means and variances across twin order and zygosity | 4 | 18613.12 | 6658 | 5297.11 | 2.18 | 6 | 0.903 |
| ACE model | 4 | 18613.12 | 6658 | 5297.11 | 2.18 | 6 | 0.903 |
| AE model | 3 | 18613.17 | 6659 | 5295.17 | -3.64e-11 | 1 | 1.000 |
| CE model | 3 | 18652.13 | 6659 | 5334.13 | 0.39 | 1 | 4.32e-10 |

***Note:*** ep= estimated parameters; -2LL= -2 log-likelihood; df= degrees of freedom; AIC= Akaike’s information criteria; **∆**LL= change in log-likelihood; **∆**df= change in degrees of freedom. The AE model fit the data best. Estimated parameters in the full model: 4 means (MZ twin 1 mean, MZ twin 2 mean, DZ twin 1 mean, DZ twin 2 mean), 4 standard deviations ‘SD’ (MZ twin 1 SD, MZ twin 2 SD, DZ twin 1SD, DZ twin 2 SD), 2 within twin correlations (MZ and DZ). This Saturated model is then constrained across twin means, variances, twin order and zygosity (4 estimated parameters) to test whether twin modelling assumptions have been met.

**Table S7.** Univariate model fitting results for university quality independent of entrance exam achievement

| **Model** | **ep** | **-2LL** | **df** | **AIC** | **∆ LL** | **∆ df** | ***p*** |
| --- | --- | --- | --- | --- | --- | --- | --- |
| 1. Full saturated | 10 | 32893.71 | 11798 | 9297.71 | - | - | - |
| 2. Constrained means across twin order | 8 | 32893.71 | 11800 | 9293.71 | 5.44e-08 | 2 | 1.000 |
| 3. Constrained means and variances across twin order | 6 | 32893.71 | 11802 | 9289.7131 | -2.62e-10 | 4 | 1.000 |
| 4. Constrained means and variances across twin order and zygosity | 4 | 32898.52 | 11804 | 9290.52 | 4.81 | 6 | 0.569 |
| ACE model | 4 | 32898.52 | 11804 | 9290.52 | 4.81 | 6 | 0.569 |
| AE model | 3 | 32898.93 | 11805 | 9288.93 | 0.41 | 1 | 0.520 |
| CE model | 3 | 32979.92 | 11805 | 9369.92 | 81.41 | 1 | 1.84e-19 |

***Note:*** ep= estimated parameters; -2LL= -2 log-likelihood; df= degrees of freedom; AIC= Akaike’s information criteria; **∆**LL= change in log-likelihood; **∆**df= change in degrees of freedom. The AE model fit the data best. Estimated parameters in the full model: 4 means (MZ twin 1 mean, MZ twin 2 mean, DZ twin 1 mean, DZ twin 2 mean), 4 standard deviations ‘SD’ (MZ twin 1 SD, MZ twin 2 SD, DZ twin 1SD, DZ twin 2 SD), 2 within twin correlations (MZ and DZ). This Saturated model is then constrained across twin means, variances, twin order and zygosity (4 estimated parameters) to test whether twin modelling assumptions have been met.

**Table S8.** Liability threshold model fitting results for university enrolment

| **Model** | **ep** | **-2LL** | **df** | **AIC** | **∆ LL** | **∆ df** | ***p*** |
| --- | --- | --- | --- | --- | --- | --- | --- |
| Saturated model | 6 | 26712.83 | 21304 | -15895.17 | - | - | - |
| Comparison with Sub 1 model | 4 | 26712.83 | 21306 | -15899.17 | 2.10x10^-4^ | 2 | 0.99 |
| Comparison with Sub 2 model | 3 | 26712.93 | 21307 | -15901.07 | 9.96x10^-2^ | 3 | 0.99 |

**Note:** Sub 1= constrained model equating thresholds across Twin 1 and Twin 2 within zygosity groups; Sub 2 = constrained model equating Thresholds across Twin 1 and Twin 2 and zygosity group; ep= estimated parameters; -2LL= -2 log-likelihood; df= degrees of freedom; AIC= Akaike’s information criteria; **∆**LL= change in log-likelihood; **∆**df= change in degrees of freedom.

**Table S9**. Twin *ACE* estimates and 95% confidence intervals indicating the proportion of variance in university success variables attributable to genetic and environmental factors.

|  | **A** | **C** | **E** |
| --- | --- | --- | --- |
| Entrance exam achievement | 0.57 (0.52-0.63) | 0.12 (0.07-0.17) | 0.31 (0.30-0.33) |
| University enrolment | 0.51 (0.44-0.58) | 0.36 (0.29-0.42) | 0.13 (0.11-0.15) |
| University quality (total score) | 0.57 (0.49-0.65) | 0.08 (0.01-0.14) | 0.35 (0.32-0.37) |
| University achievement | 0.46 (0.33-0.52) | 0.00 (0.00-0.11) | 0.53 (0.49-0.58) |
| University quality regressed for entrance exam achievement | 0.47 (0.37-0.54) | 0.03 (0.00-0.10) | 0.50 (0.47-0.54) |

**Note:** A= Additive genetic influence, C= Common (shared) environmental influence, E= Non-shared environmental influence. If confidence intervals include 0 then the estimate was not significant.

**Table S10.** Multivariate model fitting results for university entrance exam achievement, university quality and university achievement. Comparing correlated factors, independent pathway and common pathway models.

| **Model** | **ep** | **-2LL** | **df** | **AIC** | **∆ LL** | **∆ df** | ***p*** |
| --- | --- | --- | --- | --- | --- | --- | --- |
| Full saturated | 54 | 100856.0 | 38700 | 23455.98 | - | - | - |
| Correlated factors | 21 | 100872.7 | 38733 | 23406.75 | 16.76 | 33 | 0.99 |
| Independent pathway | 21 | 100880.5 | 38733 | 23414.50 | 24.52 | 33 | 0.86 |
| Common pathway | 18 | 100921.1 | 38737 | 23447.13 | 65.15 | 37 | 0.002 |

***Note:*** ep= estimated parameters; -2LL= -2 log-likelihood; df= degrees of freedom; AIC= Akaike’s information criteria; **∆**LL= change in log-likelihood; **∆**df= change in degrees of freedom. The correlated factors solution fit the data best.

**Table S11.** Genetic, shared environmental and non-shared environmental correlations between university success measures

a. Genetic correlations between university success variables

|  | **Entrance_achiev.** | **Uni_Quality** | **Uni_Achiev.** |
| --- | --- | --- | --- |
| **Entrance_achiev.** | 1.00 |  |  |
| **Uni_Quality** | 0.76 | 1.00 |  |
| **Uni_Achiev.** | 0.49 | 0.27 | 1.00 |

b. Shared environmental correlations between university success variables

|  | **Entrance_achiev.** | **Uni_Quality** | **Uni_Achiev.** |
| --- | --- | --- | --- |
| **Entrance_achiev.** | 1.00 |  |  |
| **Uni_Quality** | 0.81 | 1.00 |  |
| **Uni_Achiev.** | 0.35 | 0.27 | 1.00 |

c. Non-shared environmental correlations between university success variables

|  | **Entrance_achiev.** | **Uni_Quality** | **Uni_Achiev.** |
| --- | --- | --- | --- |
| **Entrance_achiev.** | 1.00 |  |  |
| **Uni_Quality** | 0.35 | 1.00 |  |
| **Uni_Achiev.** | 0.03 | 0.09 | 1.00 |

**Table S12.** Polygenic score prediction (R²) of university success variables with 95% confidence intervals

|  | R² | 95% CIs | |
| --- | --- | --- | --- |
| Entrance exam achievement | .041 | .028 | .054 |
| University attainment | .053^a^ | .039 | .067 |
| University quality | .023 | .010 | .035 |
| University achievement | .007 | -.002 | .016 |

***Note:*** CIs = confidence intervals; ^a^= Variable was dichotomous, therefore Nagelkerke R² was calculated.

**Table S13.** Sample sizes and EduYears GPS prediction (R²) for STEM and humanities subjects

|  | *N* whole | *N* GPS | STEM | Humanities |
| --- | --- | --- | --- | --- |
| natural sciences | 517 | 210 | ✓ |  |
| mathematics/statistics | 124 | 44 | ✓ |  |
| medicine/veterinary | 133 | 45 | ✓ |  |
| engineering | 139 | 50 | ✓ |  |
| technology/design | 98 | 50 | ✓ |  |
| computing/IT | 88 | 30 | ✓ |  |
| social sciences | 498 | 194 |  | ✓ |
| arts | 429 | 162 |  | ✓ |
| humanities | 404 | 167 |  | ✓ |
| languages | 96 | 36 |  | ✓ |
| law | 109 | 36 |  | ✓ |
| Total (whole sample) | 2635 |  | 1099 | 1536 |
| Total (GPS) |  | 1024 | 429 | 595 |
| GPS prediction of degree achievement (R²) |  |  | 0.003 | 0.015 |

**Note:** N whole = total sample size (individuals) with exclusions; N GPS = sample size for GPS analysis; STEM = Science, technology, engineering and mathematics. We compared the *EduYears* GPS prediction of STEM degree achievement to Humanities degree achievement using Fisher’s r-to-z transformation. We found no significant difference between the predictions (*z* = 1.08, *p* = 0.28 two tailed).

**
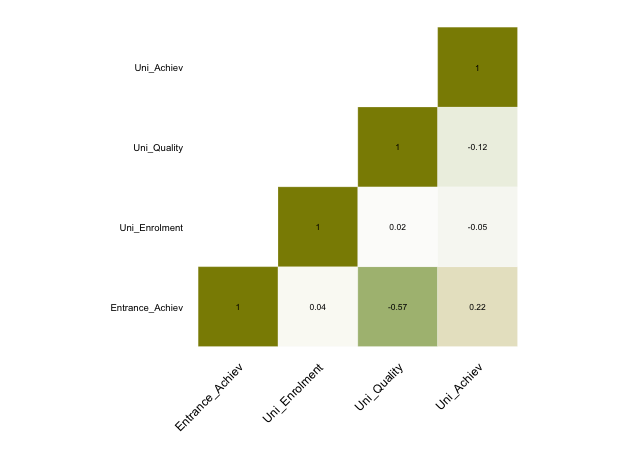
**

**Figure S1.** Correlations between each of the university success variables. ***Note:*** Entrance_Achiev= entrance exam achievement; Uni_Enrolment= university enrolment; Uni_Quality= university quality and Uni_Achiev= university achievement.

**Figure S2.** Polygenic score prediction for each of the university success variables at each of *pvalue* thresholds tested using PRSice.
